# Diagnostic utility of metagenomic next-generation sequencing for tissue in patients with suspected infectious diseases

**DOI:** 10.3389/fcimb.2025.1634406

**Published:** 2025-11-25

**Authors:** Yaqing Liu, Yuting Tan, Fei Xia, Songjie Wu, Shi Zou, Qianhui Chen, Jie Liu, Shihui Song, Qian Du, Wei Guo, Ke Liang

**Affiliations:** 1 Department of Infectious Diseases, Zhongnan Hospital of Wuhan University, Wuhan, China; 2 Wuhan Research Center for Infectious Diseases and Cancer, Chinese Academy of Medical Sciences, Wuhan, China; 3 Department of Pharmacy, Hubei No.3 People’s Hospital of Jianghan University, Wuhan, China; 4 Department of Pathology, Zhongnan Hospital of Wuhan University, Wuhan, China; 5 Department of Pathology, School of Basic Medical Sciences, Wuhan University, Wuhan, China; 6 Department of Nosocomial Infection Management, Zhongnan Hospital of Wuhan University, Wuhan, China; 7 Hubei Engineering Center for Infectious Disease Prevention, Control and Treatment, Wuhan, China

**Keywords:** metagenomic, next-generation, sequencing, tissue, conventional, microbiological tests, consistency, sensitivity

## Abstract

Metagenomic next-generation sequencing (mNGS) was suggested to potentially replace traditional microbiological methods because of its comprehensiveness. However, the diagnostic utility of mNGS for tissue hasn’t been fully explored, especially for patient with HIV infection. HIV-positive and negative patients with suspected infectious diseases who performed tissue mNGS and conventional microbiological tests (CMTs) were retrospectively enrolled between October, 2020 and May 2024. The microbial spectrum of tissue mNGS and CMTs was analyzed, and the diagnostic accuracy and consistency of mNGS and CMTs for tissue were compared. The related factors of positive rate of mNGS was analyzed. Of 70 patients with suspected infectious diseases, 44 cases were confirmed with the infectious diseases. Among 44 patients with infectious diseases, aerobic bacteria (36.4%) was the most common detected pathogen, followed by mycobacterium tuberculosis (MTB, 18.2%), non-tuberculous mycobacteria (NTM, 13.6%) and fungus (11.4%). The sensitivity of tissue mNGS (72.7%, 95%CI 56.9%-84.5%) was significantly higher than that that in tissue CMTs (29.5%, 95%CI 17.2%-45.4%) (p<0.001), but the specificity was not statistically significant(P = 0.656). mNGS demonstrated higher detection rates than CMTs in the case with single microbial infections (70.0% vs. 30.0%; p<0.01). For the case with multiple microbial infections, the detection rates of mNGS and CMTs was 100.0% and 25.5% (p=1.000), respectively. Both positive mNGS and CMTs were observed in 22.7% patients with infectious diseases, and sole positive mNGS and sole positive CMTs were observed in 50.0% and 6.8% patients, respectively. There were no statistically differences in age, gender, HIV infection, PCT levels, neutrophil counts, CD4^+^ lymphocyte count and antibiotic exposure between mNGS positive and mNGS negative groups (P > 0.05). Tissue mNGS could provide a higher sensitivity, more robust and broader method for pathogen identification by comparison with CMTs. However, CMTs shouldn’t be ignored since the low consistency between CMTs and mNGS.

## Introduction

The expanding spectrum of pathogenic microorganisms and the rising incidence of challenging-to-diagnose infections have significantly complicated etiological identification in clinical practice. The conventional microbiological tests(CMTs), including staining, culture, and polymerase chain reaction (PCR), etc, currently fail to detect pathogens in up to 60% of infectious disease cases (Schlaberg et al., 2017). Such diagnostic delays critically impede timely antimicrobial stewardship, potentially exacerbating patient outcomes.

Metagenomic next-generation sequencing (mNGS) has emerged as a transformative diagnostic paradigm, offering distinct advantages over conventional approaches. By enabling hypothesis-free detection of all microbial genetic material (bacteria, virus, fungus, and parasite) within 24–48 hours, this high-throughput technology demonstrates particular utility in complex infection scenarios (Miao et al., 2018; Qian et al., 2020; Duan et al., 2021). While existing validation studies have primarily focused on liquid biospecimens (blood, bronchoalveolar lavage fluid, cerebrospinal fluid, etc) or single tissue samples (Miller et al., 2019; Jin et al., 2022; Sun et al., 2022; Dong et al., 2024), a critical knowledge gap persists regarding mNGS performance in a such large-scale investigation for diverse tissue samples.

To address this unmet need, we conducted a study to investigate the diagnostic accuracy and consistency of mNGS and CMTs in the tissue sample. Our findings establish a reference for optimizing infection diagnosis and antimicrobial decision-making in scenarios where conventional diagnostics prove inadequate.

## Methods

### Study participants

The hospitalized patients with suspected infectious diseases in Zhongnan Hospital who performed tissue mNGS and CMTs at the same time were retrospectively enrolled from October 2020 to May 2024. The inclusion criteria included: 1) clinical evidences of infectious diseases existed, including fever, elevated white blood cell (WBC) count and/or elevated serum inflammatory markers; 2) both mNGS and CMTs (including staining, culture, PCR, Geen/Xpert, etc) of tissue were performed during hospitalization.

### Data collection

Clinical data were retrospectively collected from the medical records of Zhongnan Hospital. These following data were extracted: age, gender, HIV infection status, collection date, type of tissue sample, antibiotic use within 3 months, immunosuppressive therapy (glucocorticoids and immunosuppressants) within 3 months, lymphocyte count, CMTs and mNGS results of tissue samples.

### Positive criteria of tissue mNGS

The criteria for the positive result of tissue mNGS referred to previous studies (Miao et al., 2018; Tan et al., 2023; Chen et al., 2024; Zou et al., 2024): (1) Bacteria (excluding mycobacteria), viruses, and parasites: mNGS identified a microbe at the species level with a coverage rate that was 10-fold greater than that of any other microbes. (2) Fungi: mNGS identified a microbe at the species level with a coverage rate that was 5-fold higher than that of any other fungus, attributed to its low biomass during DNA extraction. (3) Mycobacteria: Mycobacterium tuberculosis (MTB) was deemed positive when at least one read was mapped to either the species or genus level, owing to the challenges associated with DNA extraction and the low risk of contamination. Non-tuberculous mycobacteria (NTM) were classified as positive when the number of mapping reads (at the genus or species level) ranked among the top 10 in the bacterial list, reflecting the balance between hospital-to-laboratory environmental contamination and low yield rates. (4) The identification of pathogenicity is based on the patient’s clinical manifestations, immune status, underlying diseases, imaging results, mNGS findings, other auxiliary examinations, response to antibiotic treatment, and the assessments of clinical experts.

### CMTs methods

1.Staining Methods: Gram staining for bacteria (aerobic/anaerobic) and fungal hyphae; Acid-fast staining (Ziehl-Neelsen) for mycobacteria (MTB/NTM); Gomori methenamine silver (GMS) and Periodic Acid-Schiff (PAS) staining for fungi; Histopathological examination (H&E staining) for tissue architecture assessment. Immunohistochemistry (IHC) employed pathogen-specific monoclonal antibodies on tissue sections via automated staining (Leica Bond RX; polymer-HRP/DAB). 2.Culture Techniques: Aerobic cultures: Blood agar and MacConkey agar incubated at 35°C for 48–72 hours; Anaerobic cultures: Brucella agar with vitamin K1/hemin, incubated anaerobically for 5–7 days; Mycobacterial cultures: Löwenstein-Jensen medium and MGIT 960 system (Becton Dickinson), incubated for 6–8 weeks. Fungal cultures: Sabouraud dextrose agar incubated at 30°C for 4 weeks. 3. Molecular Assays: PCR Targets: 16S rRNA gene for bacteria, ITS for fungi, and rpoB gene for MTB/NTM. GeneXpert MTB/RIF Assay: For MTB detection and rifampin resistance screening.

### Statistical analysis

SPSS 27.0 and GraphPad Prism 10.0 were used for the statistical analyses and plotting. Kolmogorov-Smirnov test was used to evaluate whether the measurement data conformed to the normal distribution. Continuous variables that conformed to the normal distribution were analyzed by group t-test, and vice were analyzed by non-parametric rank sum test. Comparative analysis was conducted by Pearson χ^2^ test. P < 0.05 was considered significant.

### Ethics

This study was approved by the Ethics Committee of Zhongnan Hospital of Wuhan University (2024310K).

## Results

### Patients’ characteristics

Among 70 patients with suspected infectious diseases, 6 patients were finally excluded for undefined diagnosis. Total 44 cases were diagnosed with infectious diseases (ID), and 20 cases were diagnosed with no-infectious diseases (NID) were enrolled in the study. Among the enrolled patients, 32(50.0%)were HIV positive, and 25(57.8%) were HIV negative. There was no significant difference between ID group and NID group in age, HIV status, sex, antibiotic using within 3 months, immunosuppressive therapy within 3 months, tissue type. The most of tissue samples were obtained from lymph nodes, followed by lung, skin and subcutaneous lump (**Table 1**).

**Table 1:** Characteristics of the enrolled patients (N = 64).

Characteristics	ID group(n=44)	NID group(n=20)	P value
Demographic and cliniclal data			
Age[years]	46.07 ± 15.50	53.50 ± 15.98	0.083
Male, n (%)	35(79.5)	17(85.0)	0.863
HIV infection, (n)%	25(57.8)	7(35.0)	0.106
Antibiotic using within 3 months, n (%)	27(61.4)	9(45.0)	0.221
Immunosuppressive therapy within 3 Months, n (%)	2(4.5)	2(10.0)	0.781
Tissue sample site, n (%)			
Lymph node	13(29.5)	6(30.0)	0.164
Lung	8(18.2)	5(25.0)	
Skin and subcutaneous lump	8(18.2)	1(5.0)	
Centrum and intervertebral disc	7(15.9)	0	
Liver	2(4.5)	3(15.0)	
Gastrointestinal tract	2(4.5)	1(5.0)	
Cerebrum	0	2 (10)	
Oral cavity	1(2.3)	1(5)	
Bone	0	1(5)	
Synovium	1(2.3)	0	
Maxillary sinus	1(2.3)	0	
Rectrobulbar tissue	1(2.3)	0	

### Pathogens spectrum among ID group

**Figure 1:**
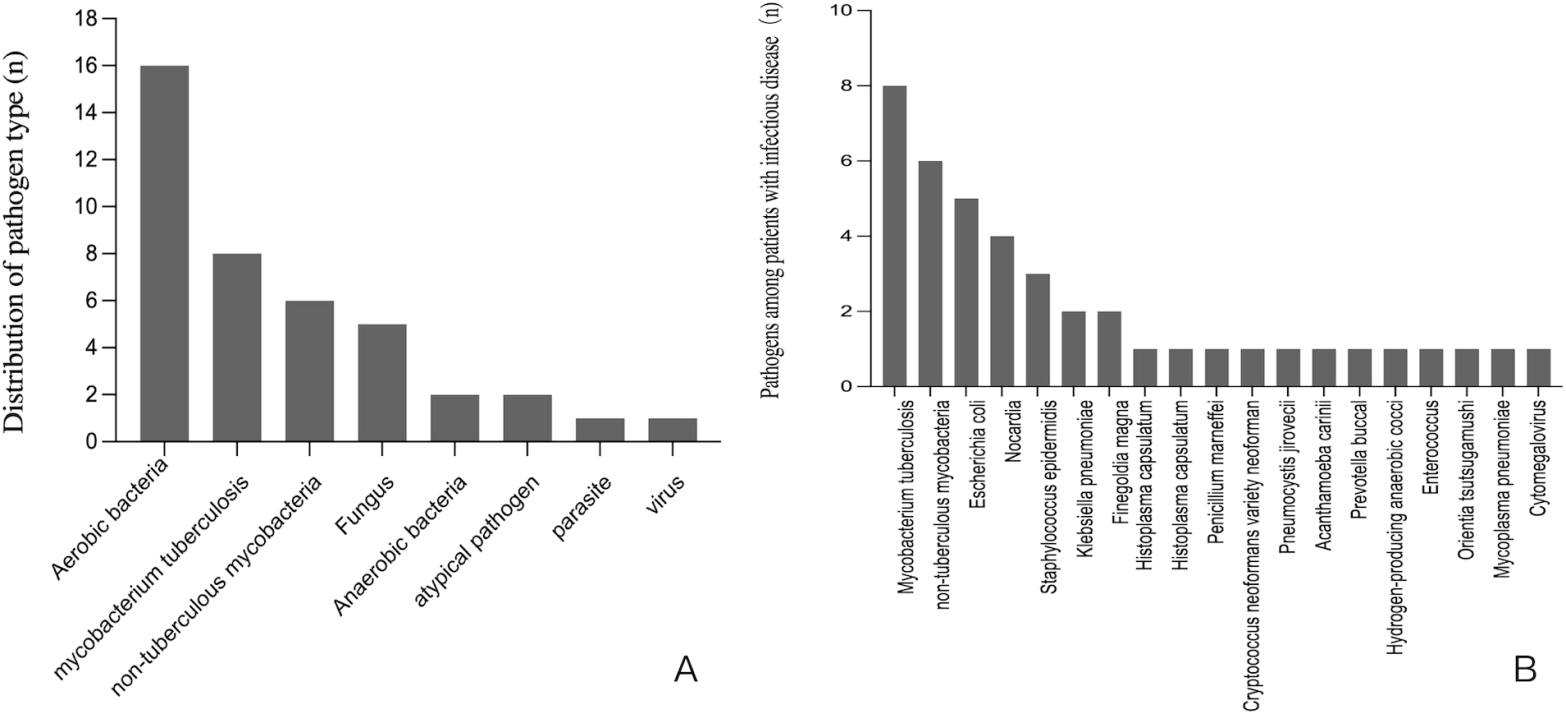
**(A)** The distribution of pathogen type among patients with infectious disease. **(B)** The pathogens spectrum among 44 patients with infectious disease.

Among 44 patients ultimately diagnosed with infectious diseases through tissue-based mNGS and/or CMTs, 72.7% were HIV-positive. The distribution of pathogen type was shown in **Figure 1A**. Aerobic bacteria (n=16) was the most frequently detected, followed MTB (n=8), NTM (n=6), fungus (n=5), anaerobic bacteria (n=2), atypical pathogen (n=2), parasite (n=1) and virus (n=1). As shown in **Figure 1B**, for pathogens spectrum of 44 patients with infection, MTB (n=8) was the most common detected pathogen, followed by NTM (n=6), *Escherichia coli* (n=5), *Nocardia* (n=4), *Staphylococcus epidermidis* (n=3), *Klebsiella pneumoniae* (n=2) and *Finegoldia* magna(n=2).

In ID group, there were 40 cases (90.9%) diagnosed with monomicrobial infection and 4 cases (9.1%, all are HIV positive) diagnosed with polymicrobial infection (≥2 non-HIV pathogens). Among 40 patients with monomicrobial infection, 12 cases (30.0%) were detected via CMTs, while 28 cases (70.0%) were detected by mNGS. Among 4 polymicrobial infections, 1 case (25.5%) were detected via CMTs, while 4 cases (100.0%) were detected by mNGS. For monomicrobial infection, the detection rate of mNGS was higher than that of CMTs (p <0.001).

### Consistency between mNGS and CMTs in ID group

**Figure 2:**
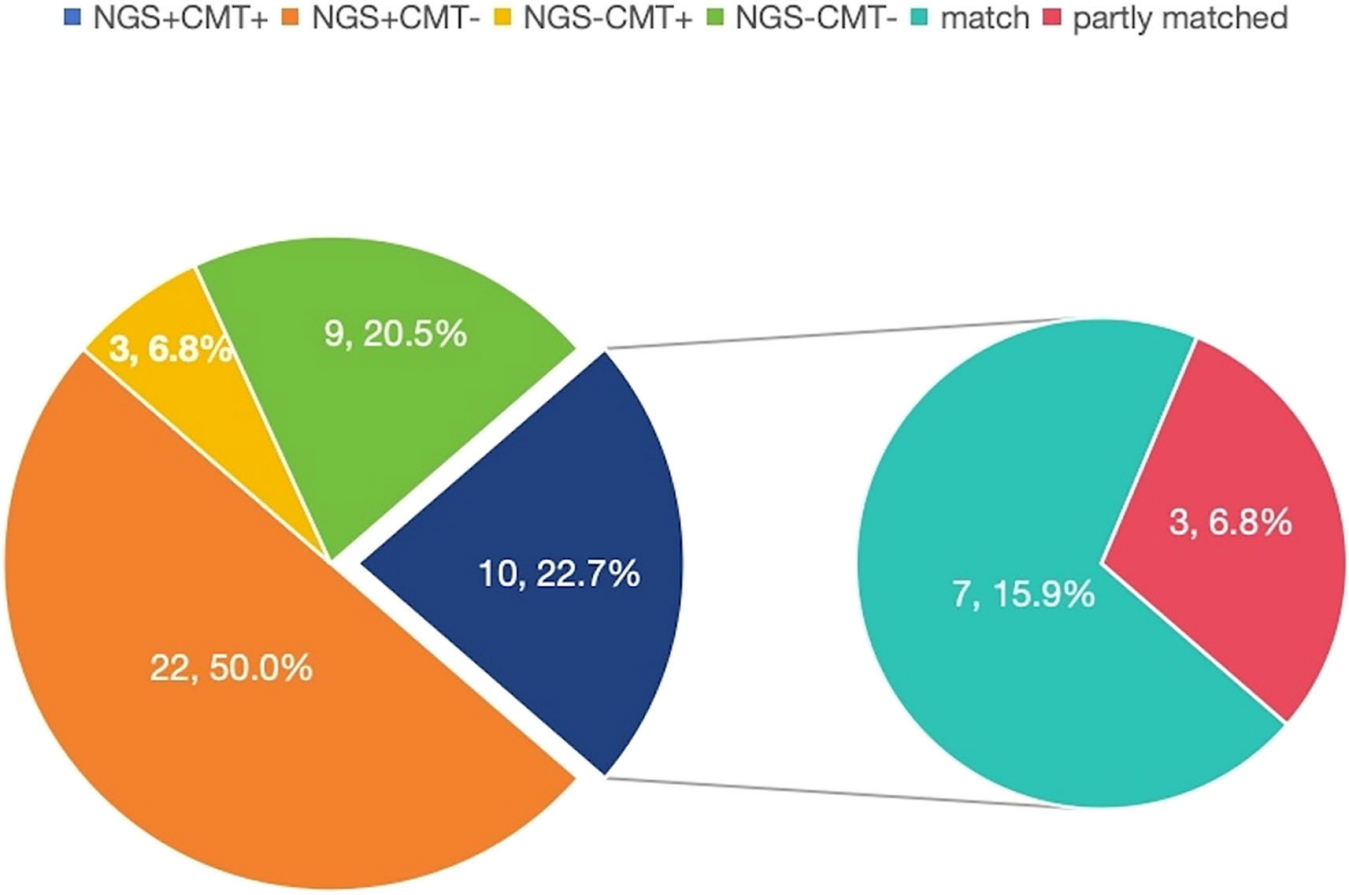
The consistency between tissue mNGS and CMTs in patients with infectious disease.

In ID group, both positive mNGS and CMTs were observed in 10 (22.7%) patients, and both negative mNGS and CMTs were observed in 9 (20.5%) patients. Sole positive mNGS and sole positive CMTs were observed in 22 (50.0%) and 3 (6.8%) patients, respectively. For double-positive samples, the 2 results were completely matched in 7 cases (**Figure 2**).

### Comparison of diagnostic performance between mNGS and CMTs

**Figure 3:**
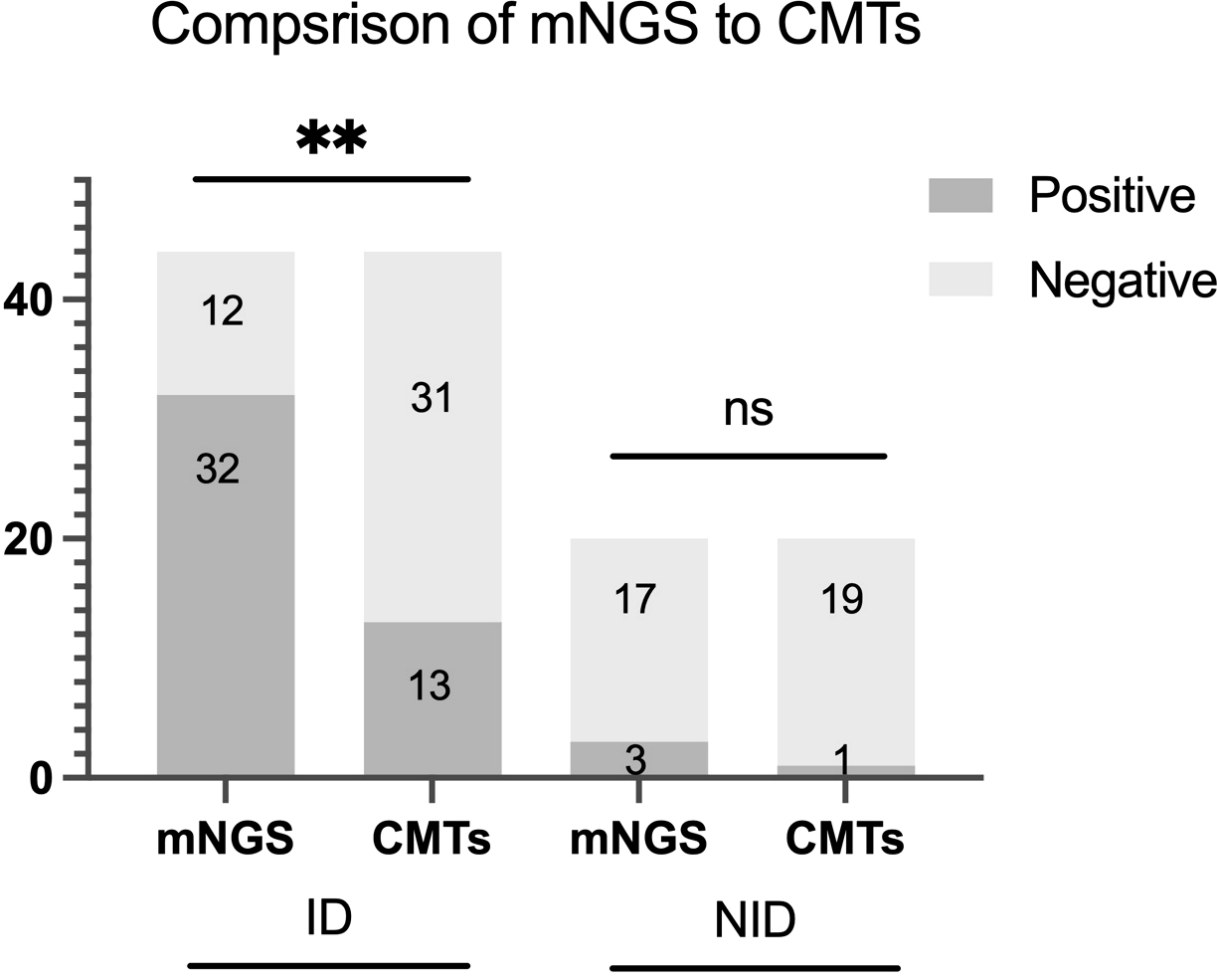
Positive rate comparison and concordance analysis between metagenomic next-generation sequencing (mNGS) and conventional microbiological tests (CMTs) for infectious disease (ID) group and noninfectious disease (NID) group. There were statistical differences between mNGS and CMTs of ID group (P < 0.01), but no differences in NID group (P > 0.05). The number of positive samples (y-axis) for pairwise mNGS and CMTs testing is plotted against the ID and NID groups (x-axis). **P<0.01, ns P>0.05.

The diagnostic accuracy was further compared between mNGS and CMTs for tissue. The sensitivity, specificity, positive predictive value (PPV) and negative predictive value (NPV) of tissue mNGS were 72.7% (95%CI 56.9%-84.5%), 85.0% (95%CI 61.1%-96.0%), 91.4% (95%CI 75.8%-97.8%) and 58.6% (95%CI 39.1%-75.9%), respectively. The sensitivity, specificity, PPV and NPV of tissue CMTs were 29.5% (95%CI 17.2%-45.4%), 95.0% (95%CI 73.0%-99.7%), 92.9% (CI 64.3%-99.6%), 38% (CI 25.5%-52.8%), respectively. Compared with CMTs, the sensitivity of mNGS was significantly higher (p<0.001), but the specificity between the two methods had no significant difference (p=0.625). When combined with tissue mNGS and CMTs, the sensitivity was raised to 75.6%(p=0.250), the specificity was decreased to 78.9% (p=1.000) (**Figure 3**).

### Correlative factors for detection of mNGS

The correlative factors including demographic characteristics, inflammatory and immune function indicators, and antibiotic exposure are analyzed. The results showed (**Table 2**) that there were no statistically differences in the average age, gender, HIV infection status, CD4^+^ lymphocyte count (CD4 count), PCT level, absolute neutrophil count, percentage of neutrophil and antibiotic exposure between positive and negative groups by mNGS (P > 0.05).

**Table 2:** The analysis of the related factors for detection of mNGS.

Characteristics	Positive (n=32)	Negative (n=12)	P value
Age, mean ± SD	48.31 ± 15.07	40.08 ± 15.04	0.118
Male, n(%)	25(78.13)	10(83.33)	1.000
HIV infection	18(56.25)	7(58.33)	1.000
CD4 cells/μL, mean ± SD	258.22 ± 263.66	285.17 ± 232.32	0.757
PCT ng/ml, [median(IQR)]	0.23(0.05,0.46)	0.48(0.06,0.48)	0.342
Neu×10^9^/L [median(IQR)]	3.00(1.80,5.53)	2.72(1.61,6.95)	0.522
Neu%[median(IQR)]	73.45(58.45,82.93)	70.10(54.0,80.00)	0.529
Antibiotic exposure, n(%)	23(71.88)	7(58.33)	0.620

procalcitonin (PCT), neutrophil counts (Neu).

## Discussion

CMTs with low positive rate are still the routine examination methods for the diagnosis of infectious diseases. Despite the high sensitivity of mNGS in the liquid samples, the diagnostic value of mNGS for tissue in infection disease was rarely reported. Our study supplemented the associated data, demonstrating that tissue mNGS could be a useful tool for identifying the pathogens due to its higher sensitivity, more robust and broader pathogen spectrum than CMTs, which might be conductive for prompt diagnosis and early treatment in infectious disease. However, tissue CMTs should also be performed since the low consistency between mNGS and CMTs.

To date, few studies have confirmed the sensitivity and specificity of tissue mNGS. Our study found that mNGS of tissue had significantly higher sensitivity by comparison with CMTs of tissue (72.7% vs. 29.5%, p<0.001), and the comparison of specificity was 85.0% vs. 95.0% (p=0.625). Studies demonstrated that mNGS exhibits significantly enhanced diagnostic sensitivity compared to CMTs when analyzing tissue and other samples for infectious pathogens (Wang et al., 2020; Duan et al., 2021; Hao et al., 2023; Qin et al., 2024; Yang et al., 2024). In addition, our results revealed no statistically significant difference in specificity between tissue mNGS and CMTs for the diagnosis of infectious diseases. Similar to our findings, studies showed that the specificity of mNGS and CMTs in tissue and other samples was not statistically different (p>0.05) (Chen et al., 2023; Pang et al., 2023; Lv et al., 2024). One study conducted a comprehensive systematic literature review and meta-analysis of mNGS outcomes in patients with unexplained fever, the results for mNGS and CMTs revealed a pooled sensitivity of 0.91 and 0.34, with a pooled specificity of 0.64 and 0.90, respectively (Marra et al., 2024). The above results indicate that mNGS demonstrates significantly enhanced diagnostic sensitivity relative to CMTs while maintaining equivalent specificity, positioning it as a valuable diagnostic modality for detecting tissue-based infections in clinical practice. Due to the limitation of sample size, we did not analyze the sensitivity and specificity of mNGS in different tissue samples, which is worth further exploration.

The culture of tissue was an important means of obtaining a clear etiological diagnosis. However, its positive rate is often influenced by factors such as bacterial load and cultivation techniques (Miao et al., 2018). A research exploring the diagnostic value of mNGS in infectious diseases, explored the correlation between the status of immune function in patients and the positive results of pathogen examination. Similar with our results, they found that no statistical significance was observed in the neutrophil count, the CD4 count and PCT level between mNGS-positive and mNGS-negative patients (Duan et al., 2021). A study found that the use of antibiotics will reduce the positive rate of mNGS, but its positive duration is significantly longer than that of conventional blood culture (Xu et al., 2025). However, in our study, the positive rate of mNGS for tissue sample was not decreased by the use of antibiotics, which also indicates the robustness of mNGS technology in the diagnosis of infectious diseases, although it was not statistically significant. The lack of significant differences in age, gender, HIV infection, PCT levels, neutrophil counts, and antibiotic exposure between the two groups suggests that these factors may not be strong predictors of mNGS positivity in this cohort and mNGS is robust in the diagnosis of pathogen infection.

Our study suggested that mNGS and CMTs of tissue had a low consistency (43.2%) in detecting pathogens in tissue sample, and the double positive rate of these two methods was just 22.70%, only 15.9% patients detected the same pathogens exactly. Consistent with our data, studies showed that the double positive rate of mNGS and traditional culture in identifying pathogens of different clinical samples in 109 patients with different infectious diseases was 19.27% (Duan et al., 2021). Another study showed that the double positive rate of mNGS and traditional diagnostic methods in identifying pathogens of different clinical samples in 372 patients with different infectious diseases was 24.2%, but the double positive rate of diverse tissue mNGS hasn’t been studied (Wang et al., 2022). In addition to its high specificity and the low consistency between the two methods—which make CMTs indispensable—other unique advantages over mNGS cost-effectiveness, accessibility, and resistance profiling. The affordability and accessibility of CMTs make it as the preferred choice in clinic. CMTs also could provide the results of phenotypic antibiotic susceptibility. All these reasons demonstrated that tissue mNGS couldn’t replace tissue CMTs in patients with suspected infectious disease completely, and both the two methods should be performed in order to better identify the pathogens.

Study showed that bronchoalveolar lavage fluid mNGS was superior to CMTs in diagnosing monomicrobial infections (92% vs. 52%, p=0.000) and polymicrobial infections (66% vs. 14%, p=0.000) (Deng et al., 2022). Our study observed that the detection rate of mNGS (70.0%) was higher than that of CMTs (30.0%) in monomicrobial infection(p<0.01), and the detection rate of mNGS (100.0%) was higher than that of CMTs (25.5%) in polymicrobial infection, although the difference was not statistically significant. The marked disparity in detection rates likely reflects methodological advantages of mNGS, with the lack of significance potentially attributable to the limited sample size in this subgroup analysis. In our study, all patients with polymicrobial infection were HIV positive. It is well known that immunodeficient patients (HIV-Infected individuals, patients taking immunosuppressive agents, etc) are more susceptible to opportunistic infections and co-infection with multiple pathogens, therefore, tissue mNGS plays an crucial role in more comprehensive and sensitive pathogen detection for immunodeficient patients.

Our study revealed that aerobic bacteria, MTB, NTM, and fungi constituted the predominant pathogens identified. This microbial profile may be associated with two notable characteristics of our cases: a substantial proportion of HIV-positive patients (56.8%) and frequent lymph node biopsy specimens (29.5%). MTB was the most common pathogen identified by tissue mNGS, followed by NTM. Study showed people living with HIV are more likely to develop active tuberculosis than those without HIV (Adhikari et al., 2022). The clinical significance of MTB and NTM detection warrants particular emphasis. As increasingly recognized opportunistic pathogens (Cowman et al., 2019), NTM can manifest through diverse clinical presentations including pulmonary infections, pediatric cervical lymphadenitis, post-vaccination soft tissue infections, and disseminated disease in immunocompromised hosts (Daley et al., 2020; Ricotta et al., 2021). These varied manifestations underscore the critical importance of early pathogen identification and prompt therapeutic intervention. Our findings align with growing evidence highlighting the diagnostic advantages of mNGS, particularly for detecting fastidious organisms like MTB and NTM that present challenges for conventional culture-based methods. The mNGS’s capacity to overcome traditional diagnostic limitations suggests its potential value in managing complex infections, especially in immunocompromised populations.

The relative small sample size was the primary limitation of our study. Although many results demonstrated certain trends, they did not reach statistical significance. And due to the limited sample size, subgroup analyses by HIV status, tissue type or antibiotic exposure were not statistically viable, which may limit the generalizability of our findings. Therefore, future studies with expanded cohorts should validate these relationships.

## Conclusions

In summary, mNGS demonstrates superior sensitivity, more robust results and a broader pathogen detection spectrum compared to CMTs in tissue samples, particularly for fastidious organisms, as well as polymicrobial infections. mNGS should be used in combination with CMTs to optimizes diagnostic yield.

## Data Availability

The original contributions presented in the study are included in the article/supplementary material. Further inquiries can be directed to the corresponding authors.
